# Prognostic value of the Neer and Mayo-Fundacion Jimenez Diaz classifications in reverse shoulder arthroplasty for proximal humerus fractures

**DOI:** 10.1016/j.xrrt.2026.100767

**Published:** 2026-04-30

**Authors:** Akshay Daji, Devin Q. John, Chase Burzynski, Daniel F. DeShetler, Diego J.L. Lima, Howard D. Routman

**Affiliations:** a Department of Orthopaedic Surgery, HCA Florida JFK Hospital, Atlantis, FL, USA; b Nova Southeastern University, Dr. Kiran C. Patel College of Osteopathic Medicine, Fort Lauderdale, FL, USA; c Palm Beach Shoulder Service at Atlantis Orthopedics, Palm Beach Gardens, FL, USA

**Keywords:** Proximal humerus fracture, Reverse shoulder arthroplasty, Arthroplasty, Classification, Neer, Mayo-FJD, Outcomes

## Abstract

**Background:**

Reverse total shoulder arthroplasty (rTSA) is the preferred surgical treatment for non-reconstructible 7 proximal humerus fractures in the elderly population. The most common classification for 8 these fractures is the Neer classification. A more recent system is the Mayo-Fundación Jiménez 9 Díaz (Mayo-FJD) classification, which has demonstrated higher intraobserver and interobserver 10 reliability compared to the Neer system. However, their predictive utility remains unclear.

**Methods:**

The study consisted of a retrospective analysis of prospectively collected data from 40 patients treated for isolated proximal humerus fractures with rTSA by a shoulder and elbow fellowship-trained orthopedic surgeon between 2010 and 2020. Data collection included demographic information, post-operative range of motion, pain, and patient-reported outcome measures including the Simple Shoulder Test, the University of California Los Angeles score, the American Shoulder and Elbow Surgeons, and Constant Murley scores, among others. The 9 categories of the Mayo-FJD were organized into 3 groups: isolated tuberosity fractures—greater tuberosity or lesser tuberosity; humeral head compromising fractures—varus posteromedial (VPM), valgus impacted, head split (HS), head dislocation (HD), and head impression (VPM, valgus lateral, HS, HD, and head impaction); and surgical neck (SN) fractures—SN and disengaged neck (SN and DN). The *t*-test and the Kruskal-Wallis test were utilized to compare the outcomes for each fracture pattern between the 2 classifications.

**Results:**

The final cohort consisted of 13 2-part fractures (35.1%), 19 3-part fractures (51.4%), and 5 4-part fractures (13.5%) by the Neer classification, and there were 6 VPM (16.2%), 13 valgus lateral (35.1%), 3 HS (8.1%), 2 HD (5.4%), and 13 DN (35.1%) by the Mayo-FJD classification. Patients in the SN/DN group had increased pain with touching the back of their necks (*P* = .045) as well as decreased Simple Shoulder Test (*P* = .023) and University of California Los Angeles scores (*P* = .018) when compared to humeral head compromising fractures.

**Conclusion:**

Our findings suggest that fractures about the SN, specifically the DN fractures, may be associated with higher levels of pain and poorer shoulder function following rTSA. However, future studies should include a larger cohort of patients with complete subtype representation to verify our conclusion.

Reverse total shoulder arthroplasty (rTSA) has emerged as the preferred treatment option for non-reconstructible proximal humerus fractures (PHFs) in the elderly population.^1^^,^^4^^,^^10^^,^^14^ Classifying PHFs in a clinically useful way poses a considerable challenge due to the complexity and highly variable nature of these fractures. The Neer classification is the most widely used classification system, appreciated for its simplicity but with only acceptable intraobserver and interobserver agreement.^3^^,^^17^

In response to the limitations of the Neer classification, the Mayo-Fundación Jiménez Díaz (Mayo-FJD) classification system was developed in 2023 and was based on the foundational work performed by Foruria et al in 2011 and 2022 that describes PHF patterns.^5^, ^6^, ^7^, ^8^ The system categorizes PHFs based on their anatomical and biomechanical fracture patterns with 9 fracture patterns: isolated tuberosities fractures—greater tuberosity (GT) and lesser tuberosity (LT); humeral head compromising fractures—varus posteromedial (VPM), valgus lateral (VL), head split (HS), head dislocation (HD), and head impaction (HI); and surgical neck (SN) fractures—SN and disengaged neck (DN). By incorporating both the direction of deforming forces and the specific location of fracture lines, the Mayo-FJD system offers a more nuanced description of fracture morphology as compared to the Neer classification system. The Mayo-FJD classification has demonstrated superior intraobserver and interobserver reliability compared to Neer, using both radiographs and computed tomography (CT) imaging.^8^^,^^12^^,^^13^ Classification guidelines have become more stringent ever since 2011, when Kottner et al performed a study proposing guidelines on how to improve the quality of reporting.^10^ A good classification system allows for consistent communication, guides treatment, and can prognosticate outcomes. While both systems have been studied for classification reliability, their utility in predicting post-operative clinical and patient-reported outcomes following rTSA for PHFs remains unclear.

This study aimed to assess the prognostic utility of the Neer and Mayo-FJD classification systems in patients undergoing rTSA for PHFs. Given its more detailed characterization of fracture morphology and displacement, we hypothesize that the Mayo-FJD classification may offer improved ability to predict post-operative outcomes. Clarifying this relationship may enhance surgical decision-making and patient counseling while informing future research and classification use in the setting of operative management.

## Methods

We performed a retrospective analysis of 40 patients treated with rTSA for PHF by a fellowship-trained shoulder surgeon (H.D.R.) between 2010 and 2020 by assessing the prospectively collected data. In all cases, the Equinox Fracture rTSA (Exactech, Gainesville, FL) with a medialized glenoid and lateralized onlay humerus with a 145° neck-shaft angle stem was utilized. The GT was reconstructed with FiberWire (Arthrex, Naples, FL) incorporated into the humeral stem, while the LT was not reconstructed. This study was determined to be exempt from institutional review board oversight given that it was an analysis of typical data collection from a deidentified database.

Two shoulder and elbow fellowship-trained surgeons (H.D.R. and D.J.L.L.) classified each patient according to the Neer and Mayo-FJD classifications by consensus. CT scans were not routinely available. For the Neer classification, each case was assigned a number 1-4 corresponding to the number of displaced or angulated parts in the fracture.^13^^,^^15^ The Mayo-FJD classification system was organized into 3 main groups: isolated tuberosity fractures (GT and LT), humeral head compromising fractures (VPM, VL, HS, HD, and HI), and SN fractures (SN and DN). Patients were considered separately for each classification system, so some patients were able to be included in the Mayo-FJD classification but not for Neer and vice versa due to inadequate sample size to perform statistical analysis. Fractures were included in each classification system analysis only when adequate sample size and complete patient-reported outcome measures (PROMs) were available for that specific fracture category; cases belonging to groups with a single observation or missing PROMs were excluded from the corresponding classification system analysis to permit valid statistical comparison. Guidelines for reporting reliability and agreement studies were followed.^11^

Included cases had pre-operative shoulder radiographs (anteroposterior, Grashey, and lateral views) and post-operative PROMs, range of motion (ROM), pain scores, and patient satisfaction scores with a mean follow-up of 36.5 months. ROM measurements included active forward elevation, abduction, external rotation, and internal rotation. PROMs collected were the Simple Shoulder Test (SST), the American Shoulder and Elbow Surgeons score, the Shoulder Pain and Disability Index (SPADI) score, the University of California at Los Angeles (UCLA) score, Shoulder Arthroplasty Smart score, and Constant Murley score. Exclusion criteria were incomplete radiographs, more than 2 PROM or ROM values missing, treatment other than rTSA, and polytrauma. Two patients were excluded due to lack of PROM data. One had a 2-part SN fracture while the other had a 3-part HD fracture, leaving 38 patients for final analysis. An exception was made for the 4-part Neer group due to 4 of the 5 fractures missing values on CONSTANT and SPADI PROMs. Instead of excluding the 4-part fracture pattern from our entire analysis, we only excluded them from those PROMs. Subsequently, this means we also excluded the same patients’ Mayo-FJD classifications for only SPADI and CONSTANT PROMs, which were 1 VPM, 1 HD, and 2 DN.

Post-operative ROM and PROMs at final follow-up were analyzed. All data were compiled in Microsoft Excel 2016 version 2402 (©Microsoft 2025), and all statistical analyses were performed using R (version 4.5.1; R Foundation for Statistical Computing, Vienna, Austria). Fisher's exact test was used to compare gender distribution between groups, *t*-test was used to compare the age and outcomes for the groups in the Mayo-FJD comparison and Kruskal-Wallis test was used for the Neer comparison. The following questions were analyzed: (1) Do the patients classified as Neer 3 or 4 have worse outcomes than those classified as 1 or 2? (2) Do any groups of the Mayo-FJD classification have worse outcomes compared to the others? (3) Were those groups within the Mayo-FJD classification who had worse outcomes classified as Neer 3 or 4? The primary outcome of this study was to determine if the Mayo-FJD classification system provided a better prognostic value than the Neer classification system for PROMs following PHFs treated with rTSA.

## Results

The Mayo-FJD system and Neer system both classified a total of 37 patients. The cohort had a mean age of 73.9 years at the time of surgery, and most patients were female (83.8%). There was no statistically significant difference between the groups in either comparison (Tables I and II).

**Table I:** Demographics for patients included in the Neer analysis∗.

Variable	2-part(N = 13)	3-part(N = 19)	4-part(N = 5)	*P* value
Age at surgery	73.3 ± 7.7	73.4 ± 9.4	79.0 ± 4.9	.468
Gender				1.000
Male	2 (15.4%)	3 (15.8%)	1 (20%)	
Female	11 (84.6%)	16 (84.2%)	4 (80%)	

∗ Values are presented as the mean ± standard deviation or as the number of patients (% within the group) for all patients.

**Table II:** Demographics for patients included in the Mayo-FJD analysis∗.

Variable	HH Fx (N = 24)	SN Fx (N = 13)	*P* value
Age at surgery	73.6 ± 9.1	74.8 ± 7.4	.661
Gender			.391
Male	3 (12.5%)	3 (23%)	
Female	21 (87.5%)	10 (77%)	

*HH*, humeral head; *SN*, surgical neck; *Mayo-FJD*, Fundación Jiménez 9 Díaz.

∗ Values are presented as the mean ± standard deviation or as the number of patients (% within the group) for all patients.

**Figure 1:**
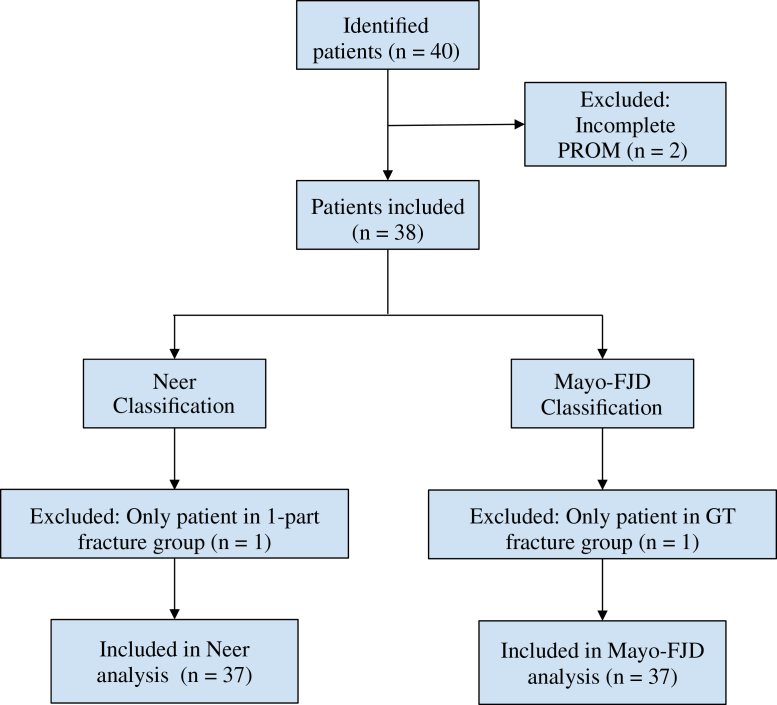
Flow diagram demonstrating patient inclusion and exclusion for both the Neer and Mayo–FJD classification analyses. *Mayo-FJD*, Fundación Jiménez 9 Díaz; *GT*, greater tuberosity.

Regarding the Neer classification, there were 13 2-part fractures (35.1%), 19 3-part fractures (51.4%), and 5 4-part fractures (13.5%). As for the Mayo-FJD classification, 6 VPM (16.2%), 13 VL (35.1%), 3 HS (8.1%), 2 HD (5.4%), and 13 DN (35.1%). There were no LT or HI type fractures in the cohort. Further categorization followed, giving 13 fractures in the SN fracture group and 24 in the humeral head compromising fracture group. Due to inadequate sample size, the one patient who was classified as a 1-part fracture group from the Neer classification and classified as a valgus impacted fracture group from the Mayo-FJD system, was excluded from only the Neer Analysis of Variance analysis. The same issue occurred with the GT fracture group, which only had 1 patient, who was also characterized as a 2-part fracture in the Neer system and was excluded only from the Mayo-FJD analysis. Two additional patients who were categorized as a 2-part and SN fracture and another classified as a 3-part and HD fracture were also excluded from analysis due to not having any available PROMs. This leaves 37 fractures to be included for both classification systems for final Analysis of Variance analysis (Fig. 1).

The Neer classification system demonstrated no significant difference (all *P* > .05) between 2-part, 3-part, and 4-part fracture groups regarding post-operative PROMs, ROM, or pain (Table III, Table IV, Table V).

**Table III:** Neer post-operative patient-reported outcome measures∗.

Variable	2-part (N = 13)	3-part (N = 19)	4-part (N = 5)	*P* value
SST	6.5 ± 4.2	7.4 ± 3.9	5.7 ± 4.7	.669
CONSTANT	63.3 ± 14.4	59.6 ± 15.9	N/A	.585
ASES	63.4 ± 29.2	72.6 ± 25.3	70.0 ± 38.2	.577
UCLA	25.9 ± 6.8	27.4 ± 6.4	22.3 ± 7.5	.398
SPADI	56.5 ± 40.8	45.8 ± 31.3	N/A	.342
SAS	62.0 ± 11.2	66.4 ± 16.6	55.7 ± 34.1	.737
Patient satisfaction ranking	1.1 ± 1.2	1.2 ± 1.0	1.6 ± 0.5	.713

*SST*, Simple Shoulder Test; *ASES*, American Shoulder and Elbow Surgeons; *UCLA*, University of California Los Angeles; *SPADI*, Shoulder Pain and Disability Index; *SAS*, Shoulder Arthroplasty Smart.

∗ Values are expressed as the mean and the standard deviation.

**Table IV:** Neer post-operative active range of motion∗.

Variable	2-part (N = 13)	3-part (N = 19)	4-part (N = 5)	*P* value
Abduction	112.3 ± 33.7	114.7 ± 32.9	80.0 ± 43.6	.255
Forward elevation	123.1 ± 41.7	132.1 ± 36.6	106.0 ± 57.7	.481
Internal rotation†	1.9 ± 1.0	2.6 ± 2.1	2.4 ± 1.3	.752
External rotation	30.0 ± 19.1	32.6 ± 19.7	22.0 ± 22.8	.667

∗ Values are expressed as the mean and the standard deviation, in degrees.

† Expressed using the standardized system for internal rotation score.

**Table V:** Neer post-operative pain scores∗.

Variable	2-part (N = 13)	3-part (N = 19)	4-part (N = 5)	*P* value
Daily basis	3.1 ± 3.4	1.8 ± 2.3	2.2 ± 1.5	.398
Worst	4.6 ± 4.1	2.6 ± 2.8	5.6 ± 2.9	.092
Lying on affected side	3.5 ± 4.3	2.7 ± 3.1	2.3 ± 2.6	.954
Touching back of the neck	4.2 ± 4.5	1.6 ± 2.3	2.6 ± 3.3	.274
Pushing with affected arm	4.2 ± 4.0	1.7 ± 2.4	2.0 ± 1.0	.109

∗ Values are expressed as the mean and the standard deviation.

On the contrary, the Mayo-FJD classification system demonstrates patients categorized in the SN (SN/DN) fracture group have worse post-operative UCLA (*P* = .018), SST (*P* = .023), and pain when touching the back of the neck (*P* = .045) when compared to humeral head compromising fractures. There was no significant difference in other PROMs or ROM (*P* > .05) (Table VI, Table VII, Table VIII).

**Table VI:** Mayo-FJD post-operative patient-reported outcome measures∗.

Variable	HH Fx (N = 24)	SN Fx (N = 13)	*P* value
SST	8.2 ± 4.1	4.9 ± 3.1	**.023**
CONSTANT	64.5 ± 15.5	53.2 ± 5.8	.052
ASES	74.8 ± 24.2	61.1 ± 29.6	.192
UCLA	28.3 ± 6.1	22.5 ± 5.8	**.018**
SPADI	40.1 ± 34.0	65.0 ± 37.7	.129
SAS	66.5 ± 14.6	59.4 ± 17.0	.249
Patient satisfaction ranking	1.3 ± 0.9	1.0 ± 1.2	.440

*HH*, humeral head; *SN*, surgical neck; *SST*, Simple Shoulder Test; *ASES*, American Shoulder and Elbow Surgeons; *UCLA*, University of California Los Angeles; SPADI, Shoulder Pain and Disability Index; *SAS*, Shoulder Arthroplasty Smart; *Mayo-FJD*, Fundación Jiménez 9 Díaz.

Significance was measured at a value of *P* < .05.

∗ Values are expressed as the mean and the standard deviation.

**Table VII:** Mayo-FJD post-operative active range of motion∗.

Variable	HH Fx (N = 24)	SN Fx (N = 13)	*P* value
Abduction	115.0 ± 32.2	96.9 ± 39.5	.171
Forward elevation	134.6 ± 37.3	109.2 ± 45.2	.098
Internal rotation†	2.1 ± 1.7	2.6 ± 1.6	.407
External rotation	32.1 ± 19.3	24.6 ± 20.7	.294

*HH*, humeral head; *SN*, surgical neck; *Mayo-FJD*, Fundación Jiménez 9 Díaz.

∗ Values are expressed as the mean and the standard deviation, in degrees.

† Expressed using the standardized system for Internal Rotation score.

**Table VIII:** Mayo-FJD post-operative pain scores∗.

Variable	HH Fx (N = 24)	SN Fx (N = 13)	*P* value
Daily basis	1.7 ± 2.1	3.4 ± 3.3	.107
Worst	2.7 ± 2.7	5.3 ± 4.1	.056
Lying on affected side	2.5 ± 3.3	3.8 ± 4.0	.374
Touching back of the neck	1.6 ± 2.8	4.4 ± 4.1	**.045**
Pushing with affected arm	2.0 ± 2.4	4.1 ± 4.3	.146

*HH*, humeral head; *SN*, surgical neck; *Mayo-FJD*, Fundación Jiménez 9 Díaz.

Significance was measured at a value of *P* < .05.

∗ Values are expressed as the mean and the standard deviation.

## Discussion

The ideal fracture classification system should have high interobserver reliability to assist in communication between providers, should guide treatment decisions, and should predict outcomes.^8^ Numerous studies have reported limited intraobserver and interobserver reliability for the Neer classification system.^2^^,^^3^^,^^7^^,^^8^^,^^12^^,^^13^ The Mayo-FDJ classification was proposed as an improvement to the Neer classification with a more nuanced characterization of various fracture morphologies. This study assessed the prognostic utility of the Neer and Mayo-FJD classification systems for post-operative ROM and PROMs following rTSA for PHFs. Our findings demonstrate that the Mayo-FJD classification system may be predictive of clinical outcomes at a minimum 2-year follow-up, specifically with pain, post-operative UCLA, and SST scores. While the Neer classification is widely familiar and the Mayo-FJD system offers a more comprehensive description of fracture morphology, only the Mayo-FJD systems demonstrated any utility in forecasting functional results after rTSA. While both systems serve descriptive and communication purposes, this study indicates that the only the Mayo-FJD classification system is a possible prognostic tool of post-operative function or PROMs after rTSA. These findings should be carefully interpreted and are exploratory until larger cohorts with the inclusion of all fracture subtypes are performed.

An important observation within the Mayo-FJD classification system is that the SN fracture group was heavily skewed, with 13 of the 14 cases included being designated as DN fractures and only 1 being a SN fracture. In addition, the SN fracture patient, which was also reported as a 2-part fracture according to the Neer classification system, lacked many PROMs, resulting in exclusion from the study. This demonstrates that our SN fracture group is solely representative of the DN fracture, making a subanalysis not possible.

The lack of prognostic correlation from the Neer classification system could suggest that the initial fracture morphology may not be a primary determinant of outcome following rTSA. The inherent biomechanical properties of rTSA relying on deltoid tension to replace rotator cuff function may mitigate the impact of variations in fracture pattern within the patient population indicated for this procedure. Surgical techniques including implant positioning, deltoid tensioning, and tuberosity management strategies may play a more dominant role. Patient-specific factors such as age, comorbidities, pre-injury functional status, and bone quality may also be contributory and are not captured by morphological classifications. In addition, any PHFs were managed nonoperatively before the rise of surgical intervention, specifically rTSA.^1^ This may have contributed to the poor prognostic ability of the Neer Classification system, but with rTSA now being highly utilized due to its ability to have better long-term outcomes, predictability, and focus on patient functionality and independence, the Mayo-FJD classification system may play a larger role in clinical practice.^1^^,^^14^^,^^16^^,^^18^^,^^19^

## Limitations

Limitations of this study include the inherent selection bias and lack of granularity from a retrospective database, which was derived from a single fellowship-trained surgeon, which may limit generalizability. Additionally, the exclusion of less common Mayo-FJD subtypes (HS, GT, HD) and the isolated tuberosity fracture group as well as the 1-part fracture group form the Neer classification, due to insufficient sample size in conjunction with missing PROM data for the 4-part Neer group warrants caution in the interpretation of these findings. Similarly, the potential “floor effect” from the generally favorable outcomes of rTSA may mask subtle prognostic differences between classifications. Additionally, the accuracy of the Mayo-FJD system could be impacted by reliance solely on plain radiographs and not pre-operative CT scans.^5^^,^^6^ Our findings are also specific to the rTSA configuration of a single surgeon. All cases involve onlay lateralized humerus, medialized glenoid, with 145-degree neck shaft angle. These findings may reflect the soft tissue balance achieved with our implant's 145° onlay design and lateralized humeral tray. Other configurations, such as 135° inlay stems, have shown improvements in external rotation in other studies.^9^ Lastly, the overall small sample size limits the power of our findings. These limitations highlight the need for larger, multi-surgeon studies encompassing the full spectrum of fracture subtypes to validate our conclusions.

Ultimately, the Mayo-FJD classification system may be a more useful prognostic tool for PHFs when managed with rTSA. However, consistent outcomes achieved with rTSA across diverse fracture types classified by the Neer system in this single-surgeon cohort may demonstrate that well-executed rTSA reconstruction may render the initial fracture classification less prognostically relevant.

## Conclusion

The Mayo-FJD classification system demonstrated some prognostic utility for predicting post-operative PROs and pain but not ROM following rTSA for PHFs, while the Neer classification had no prognostic utility. The results suggest that the initial fracture pattern may not be the primary driver of outcomes after successful rTSA reconstruction. Surgical technique and patient factors may exert additional influence. Furthermore, future studies should include multiple surgeons and a larger cohort of patients with complete subtype representation to verify our conclusion.

## Disclaimers:

Funding: No funding was disclosed by the authors.

Conflicts of interest: Diego J. L. Lima is a consultant for Exactech. Howard D. Routman is a consultant and receives royalties from Exactech and did receive funding for the reverse total shoulder arthroplasty registry. Any additional authors, their immediate families, and any research foundations with which they are affiliated have not received any financial payments or other benefits from any commercial entity related to the subject of this article.
